# Challenges and measures during management of mounting biomedical waste in COVID-19 pandemic: an Indian approach

**DOI:** 10.1186/s42269-022-00847-4

**Published:** 2022-05-31

**Authors:** Snehal S. Manekar, Ravindrakumar L. Bakal, Rahul D. Jawarkar, Manoj S. Charde

**Affiliations:** 1 Dr. Rajendra Gode Institute of Pharmacy, University Mardi Road, Amravati, Maharashtra 444602 India; 2 Government College of Pharmacy, Karad, Maharashtra India

**Keywords:** COVID-19, Biomedical waste, Disposal, Measures, Challenges

## Abstract

**Background:**

During coronavirus pandemic, an unpredictable pile of biomedical waste (BMW) gathers at the top. India produces 710 tonnes of biomedical waste daily. The contribution of COVID-19 related biomedical waste was 126 tonnes per day in first wave of the pandemic. BMW's rapid growth is putting a strain on current waste management facilities, especially in developing countries. A sudden boost in biomedical waste needs rapid and proper segregation and disposal methods to avoid future consequences.

**Main body of the abstract:**

From literatures and statistical data available on Central Pollution Control Board (CPCB) it shows that India lags behind in large-scale sorting, collection, careful storage, transfer and disposal of bio waste. India has its own guidelines set by the CPCB to ensure the safe disposal of biomedical waste during diagnosis, treatment and quarantine of COVID-19 patients. Although there are strict guidelines for bio-waste management, many hospitals in the process of implementing them often dispose of waste in inappropriate, chaotic and indiscriminate ways due to negligence or laziness. Often, due to poor separation practices, hospital waste is mixed with general waste, resulting in harmful overall waste flow. Waste disposal handlers are not safe due to their exposure to various health risks and inadequate training in waste management. The present review sheds light on guidelines, measures, and challenges related to biomedical waste management.

**Short conclusion:**

Improper waste separation leads to improper waste disposal. Waste generation and management issues are causing daily problems as they have a profound impact on the dramatically changing global environment, including air, water and soil pollution. In addition, BMW's daily production and its processing are inversely proportional. This situation suggests that India will soon be drowning in its own garbage. The focus of this review is on the generation and disposal of biomedical waste. Based on a review of the literature, this evaluation provides a comparative picture of the current status of waste generation, national waste management strategies, and some measures to contribute to waste management and avoid future disasters.

## Background

The length of one's life is related to the amount of garbage produced. Waste is generated by a variety of activities such as households, industrial, agricultural, institutional, municipal, commercial, and so on (Vergara and Tchobanoglous 2012). In industrialized areas, the amount of waste generated is high, but the awareness about waste management is lacking (Ferronato and Torretta 2019).

Biomedical Waste (BMW) is defined as unwanted material left during the study, manufacture, or testing of human or animal diagnosis, treatment, immunization, or biologics. Bio-Medical Waste Management Rules 2016 classify the BMW into different categories such as human anatomical waste, animal waste, microbiological and bioengineering waste, sharp waste, cytotoxic waste, contaminated waste, solid waste (waste generated from single-use items other than sharp waste), liquid waste, incinerator ash and chemical waste (Datta et al. 2018, Rule 5 of Bio-medical Waste Management Rules, 2016 and 2018, 2019). Table 1 shows the sources of biomedical waste generation (Tiwari and Kadu 2013, Kalpana et al. 2016). Table 2 indicates the categories of biomedical waste.

**Table 1:** Sources of generation of biomedical waste

Patient care	Patient service	Research	Others
Hospitals (Human and Veterinary)	Dispensaries	Clinical research labs	Domestic bio waste
Maternity home	Immunization centres	Academic research project	Slaughter house
Nursing home	Blood bank	Animal research centre	Industries
Dialysis centres	Ambulance service	Microbiology lab	
Clinic	Pathology lab

**Table 2:** Categories of biomedical waste

Sr. No	Category	Examples
1	Infectious waste	Pathogenic microorganism. Inanimate objects used in autopsy, surgery and treatment of infected patients and animals contaminated with infectious blood, humor, microbes
2	Pathological waste	Blood, humor, tissue, organs, human foetuses and animal dead body
3	Sharp	Syringes, needles, scalpels, saws, infusion sets, knives, blades, broken glass etc
4	Pharmaceutical waste	Expired, unused drugs, vaccines and sera. Contaminated pharmaceutical products
5	Genotoxic waste	Contaminated vomit, urine, or faeces of patients treated with cytotoxic drugs, chemicals, and radioactive materials
6	Chemical waste	Scrap solid, liquid, or gaseous chemicals if they are toxic and harmful
7	Waste with heavy metals	Mercury, cadmium, and lead
8	Radioactive waste	The radioisotopes used in vitro & in vivo analysis, and treatment

Patient care and service produces harmful liquid and solid medical waste, the improper handling or disposal of which has life-threatening consequences (Padmanabhan and Barik 2019). The most important and first target group is healthcare professionals, especially involved in waste management that is, separation, storage, transportation, processing and disposal (Tomlin et al. 2020).

India has individual regulations and specific rules regarding the disposal of biomedical waste. To counter the daily impact, BMW management revised previous legislation and changed previous rules into strict guidelines (Kumar et al. 2017). India is lagging behind in large-scale sorting, collection, careful storage, transfer and disposal of biowaste. Waste disposal companies are exposed to a variety of health risks (Gutberlet and Nazim Uddin 2017) and are not safe for waste due to lack of waste management training (Khan et al. 2019).

## Management of biomedical waste

Solid waste management is one of the most difficult problems in the world, especially in urban areas. Apart from strict rules and regulations and proper oversight, India is still lagging behind in proper management of BMW and its risks (Javeed and Kallihal 2021). The steps to BMW's safe and scientific management are operation, separation, cutting, disinfection, internal storage, transfer, and final treatment or disposal (Acharya and Singh 2000; Bio-Medical Waste (Management and Handling) Rules 1998). Production sites are required to separate waste into recyclable and non-recyclable, dangerous and non-hazardous components.

The main steps for disposing of medical waste are: (1) Divide into various components and store safely in suitable containers. (2) Waste disposal and move to landfill, (3) treat, (4) finally destroy. (Chand et al. 2021) To make it easier to identify the categories of medical waste, classify the waste into color-coded plastic bags or bottles (Rule 16 of Bio-medical Waste Management Rules 2016).

**Fig. 1:**
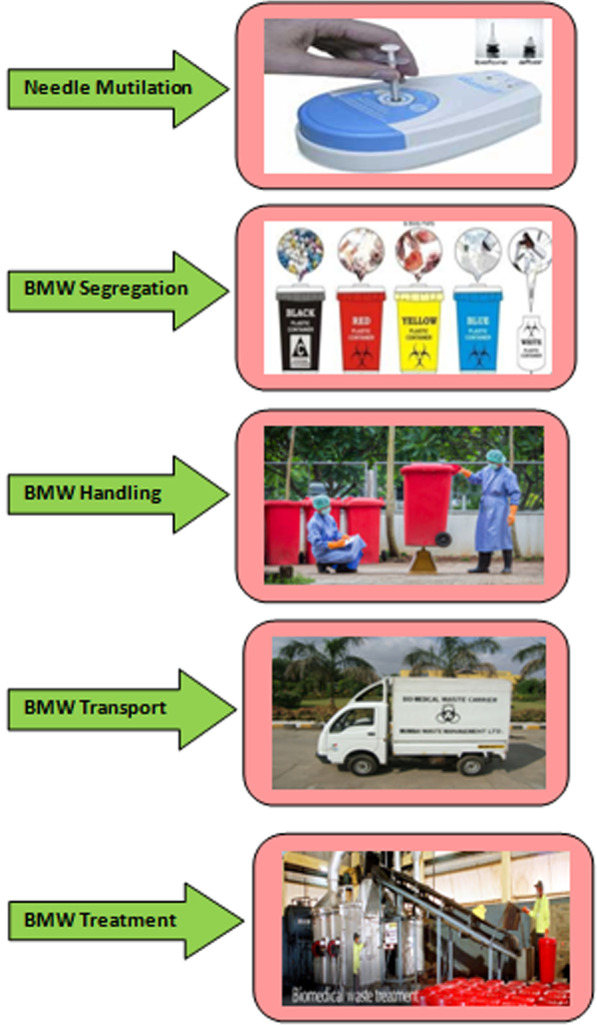
Steps involved in biomedical waste management. *Source*: Central Pollution Control Board (CPCB)

Figure 1 gives idea about sequential steps involved in biomedical waste management Central Pollution Control Board (2020b) and Central Pollution Control Board (2020c).

Following are the common methods used for disposal of biomedical waste: (Chandrappa and Das 2012).

**Fig. 2:**
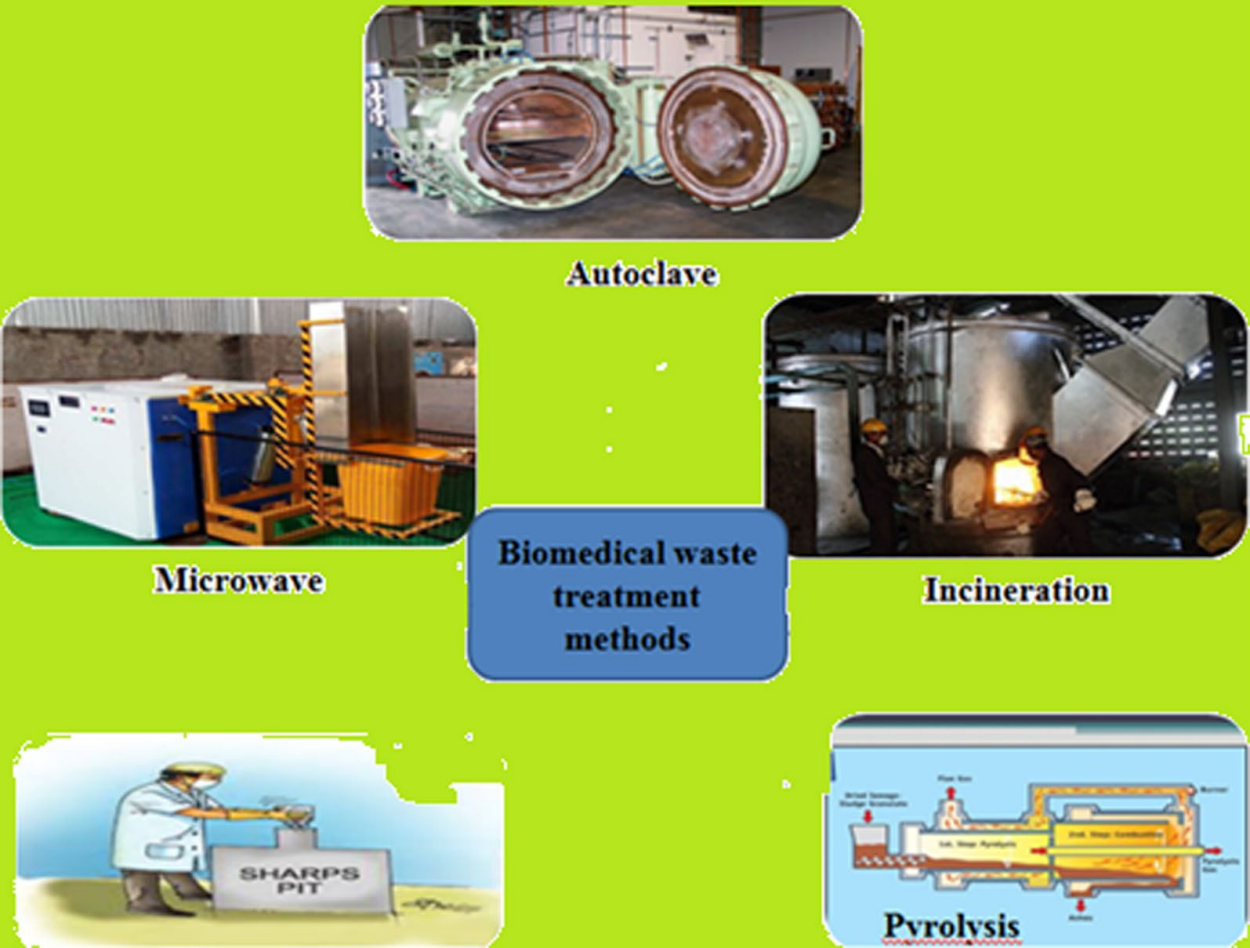
Methods of biomedical waste treatment

Figure 2 represents the different disposal methods of biomedical waste (Singh et al. 2001).

- Open dumping/ Landfill,
- Incineration/ autoclaving,
- Recycling–reuse,
- Direct use: Direct combustion/ Water streaming,
- Biological treatment: Composting, vermin composting, black soldier fly treatment, anaerobic digestion, fermentation,
- Chemical treatment: Esterification/densification.
- Thermo-chemical treatment (pyrolysis, liquefaction, gasification).

## Main text

### Sudden boost in biomedical waste in pandemic COVID-19

The invasion of the new coronavirus disease (COVID-19) has led to a surge in biomedical waste. Pressure will continue to increase as it extends and spreads its stay. Under the current conditions, the use of face masks, shields and gloves is compulsory. Under these circumstances, BMW produces more household waste in parallel or than in medical facilities (HCF). In India, 270,416 hospital supply facilities (HCF) produce 614 tonnes of biomedical waste daily (Rajak et al. 2021; CPCB 2018, 2019, Government of India website). According to information from the Central Pollution Control Board (CPCB) using the BWM application (app), the country produced 45,954 tonnes of COVID-19 waste in the year ending May 10, 2021. Since the first wave of the pandemic, the country has 126 tonnes of COVID-19 waste per day, which is about 20% of the 614 tonnes of biomedical waste produced per day. Table 3 shows amount of biomedical waste in second wave of pandemic.

**Table 3:** Biomedical waste generated during pandemic second wave. *Source*: Central Pollution Control Board

States	Total BMW in tones	%Share of COVID19 BMW
Andhra Pradesh	25	40
Arunachal Pradesh	0.5	22
Assam	9.3	6
Bihar	35.9	3
Chhattisgarh	6.5	42
Goa	1.9	23
Gujarat	58.4	38
Haryana	27.9	47
Himachal Pradesh	5.7	40
Jharkhand	7.8	7
Karnataka	94.5	18
Kerala	66.6	36
Madhya Pradesh	25.2	29
Maharashtra	81.3	23
Manipur	1.1	12
Meghalaya	1.5	17
Mizoram	1	3
Nagaland	0.7	11
Odisha	24.6	27
Punjab	20.1	20
Rajasthan	25.7	19
Sikkim	0.5	3
Tamil Nadu	71.8	19
Telangana	25.4	20
Tripura	1.42	1
Uttar Pradesh	68.4	23
Uttarakhand	5.8	34
West Bengal	47.3	12
Andaman and Nicobar Islands	0.7	2
Chandigarh	5.8	33
Dadra Nagar Haveli and Daman Diu	0.4	18
Delhi	47.6	39
Jammu and Kashmir	8.4	30
Ladakh	Not available	Not available
Lakshadweep	0.1	9
Puducherry	7.7	23

https://www.downtoearth.org.in/news/waste/covid-19-will-place-india-s-biomedical-waste-management-under-terrible-strain-77714

**Fig. 3:**
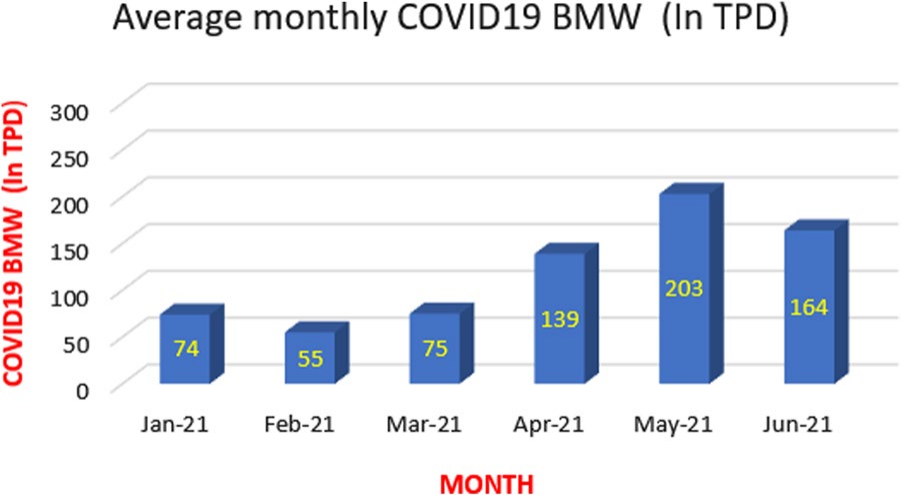
COVID-19 related bio-medical waste generation Tones/day (TPD) in India. *Source*: Central Pollution Control Board (CPCB)

Peak production in 2020 was estimated to be in the range of 180–220 tonnes / day (TPD). Since February 2021, the number of COVIDBMW generations has been gradually increasing. The Fig. 3 graph shows the average monthly generation of COVID-19 organic waste in 2021. (Manual, solid waste management).

### Hold the attention

According to CPCB data, the peak COVID-19 waste last year was September 2020. It was 183 tonnes / day, but this year's peak was recorded at 203 tonnes / day in May 2021. Therefore, the second wave of the pandemic reflects that it is more contagious than the first wave (Singh 2021).

The use of protective clothing in clinics, quarantine centers, and home quarantine facilities creates a pile of biomedical waste of concern given the already surprising increase in biomedical waste from hospitals. The 2005 Disaster Management Act described their broader approach, but did not specify that pandemics should be classified as natural disasters. When used biomedical waste from the COVID-19 Center is mixed with municipal waste and disposed of in landfills, it can lead to environmental pollution and health hazards (Gupta 2021). India is already fighting for proper BMW management and the introduction of new one-way technology. (Goswami et al. 2021) The rapid surge in BMW in a short period of time poses a major challenge in mastering the situation. In a pandemic situation, when thinking about vaccination, biomedical waste also occurs between vaccination and disease prevention (Das et al. 2021). From the start of vaccination to the present, India has reached more than 1 billion vaccinations (CSEWebnet). Vaccination produces biomedical waste such as syringes, needles and glass bottles. According to the COVID-19 vaccination instructions issued on December 28, 2020, the country has already produced approximately 1.3 billion discarded syringes and needles, and over 100 million used glass vials. Also need to be carefully disposed of. In rural India, significant numbers of vials need to be disposed of due to delays in transportation, improper storage and temperature maintenance, which lead to the additional biowaste.

### Medical waste management during covid-19 pandemic

Due to the COVID-19 outbreak in India, the Central Pollution Control Board of the Ministry of Environment, Forests and Climate has published guidelines on the disposal of waste generated during the treatment / diagnosis / quarantine of COVID-19 patients. CPCB will review the guidelines under the 2016 Biomedical Waste Management Regulations (Bio-Medical Waste Management (Amendment) Rules 2018) from time to time so that COVID-19 waste will be collected with the utmost care and transported to the "Biomedical Waste Disposal and Disposal Facility". This guide was first published on March 19, 2019 (Bio-Medical Waste Management (Amendment) Rules 2019) and then revised on July 17, 2020. In May 2020, CPCB also launched the COVID-19 Biomedical Waste Management app, a mobile application to monitor this rapidly increasing waste flow in real time. These guidelines include the use of two-layer bags, mandatory labeling of bags and containers as “COVID-19 waste", regular disinfection of special trolleys, and individual records of waste generated in the COVID-19 isolation ward. BMW will follow the 2016 administrative rules. Component of a set of personal protective equipment (PPE) like disposable masks, gloves, headgear, face shields, protective goggles, protective clothing, and shoe covers, etc. should be separated and transferred to a common facility for disposal. Nevertheless, as mentioned above, used PPE components should be stored separately for at least 72 h before being disposed of with solid waste after cutting or shredding (Amended in Rev. 2 of guidelines dated 18.04.2020 and Rev. 4 dated 17.07.2020; Criteria for 72 h is as per Centre for Disease Control & Prevention (CDC) guidelines for Decontamination and Reuse of Filtering Facepiece Respirators). Of this waste, dry disposable contents from the home (masks, headcap, etc.) can be collected as dry solid waste by the Urban Local Bodies (ULB).

The following additional measures have been taken by CPCB for improvement of management of bio-medical waste during COVID-19 pandemic (Peng et al. 2021):

i. In accordance with the 2016 Biomedical Waste Disposal Regulations, clearly color-coded bins or containers are placed in the ward to properly organize biowaste.
ii. In particular, waste from the COVID-19 isolation ward should be collected in a two-layer bag to prevent bag leakage (Indian Council of Medical Research 2020).
iii. A well-ventilated intermediate storage area must be available for separate biomedical waste before being sent for disposal and immediate disposal.
iv. A separate record of biowaste from the COVID-19 isolation ward should be kept.
v. Proper disinfection of bins and trolleys used for COVID-19 waste should be carried out daily.
vi. Medical waste-related activities at the COVID-19 ward and COVID-ICU station are reported daily to the regional SPCB and their respective common biomedical waste treatment facility (CBWTF).
vii. COVID-19 biological waste handlers and sanitation workers worked diligently to complete their tasks on schedule, allowing garbage to be collected and transported to a temporary waste storage location on time.
viii. Special yellow color bags provided by ULBs used for collection of biowaste produce from quarantine centers of COVID-19.
ix. Depending on the type of waste received at the CBWTF, it must be disposed of according to the type of waste by appropriate method (Bio-medical Waste Management Rules 2016).
x. CPCB has developed a COVID-19 waste tracking app called "COVID19BWM" to monitor biomedical waste from COVID-19. SPCBs and CBWTFs were instructed to make sure that the Tracking App was used. The CPCB issued a Show Cause Notice to 106 CBWTFs on July 21, 2020, for failing to use the COVID-19BWM tracking App.
xi. In July 2020, all SPCBs / PCCs were instructed to ensure compliance with CPCB guidelines for effective biomedical waste treatment under Sect. 5 of the Environmental Protection Act. However, CPCB has also created a separate page on its website to raise awareness of how to dispose of COVID-19 related waste, such as how to safely dispose of masks and PPE by the general public.

**Fig. 4:**
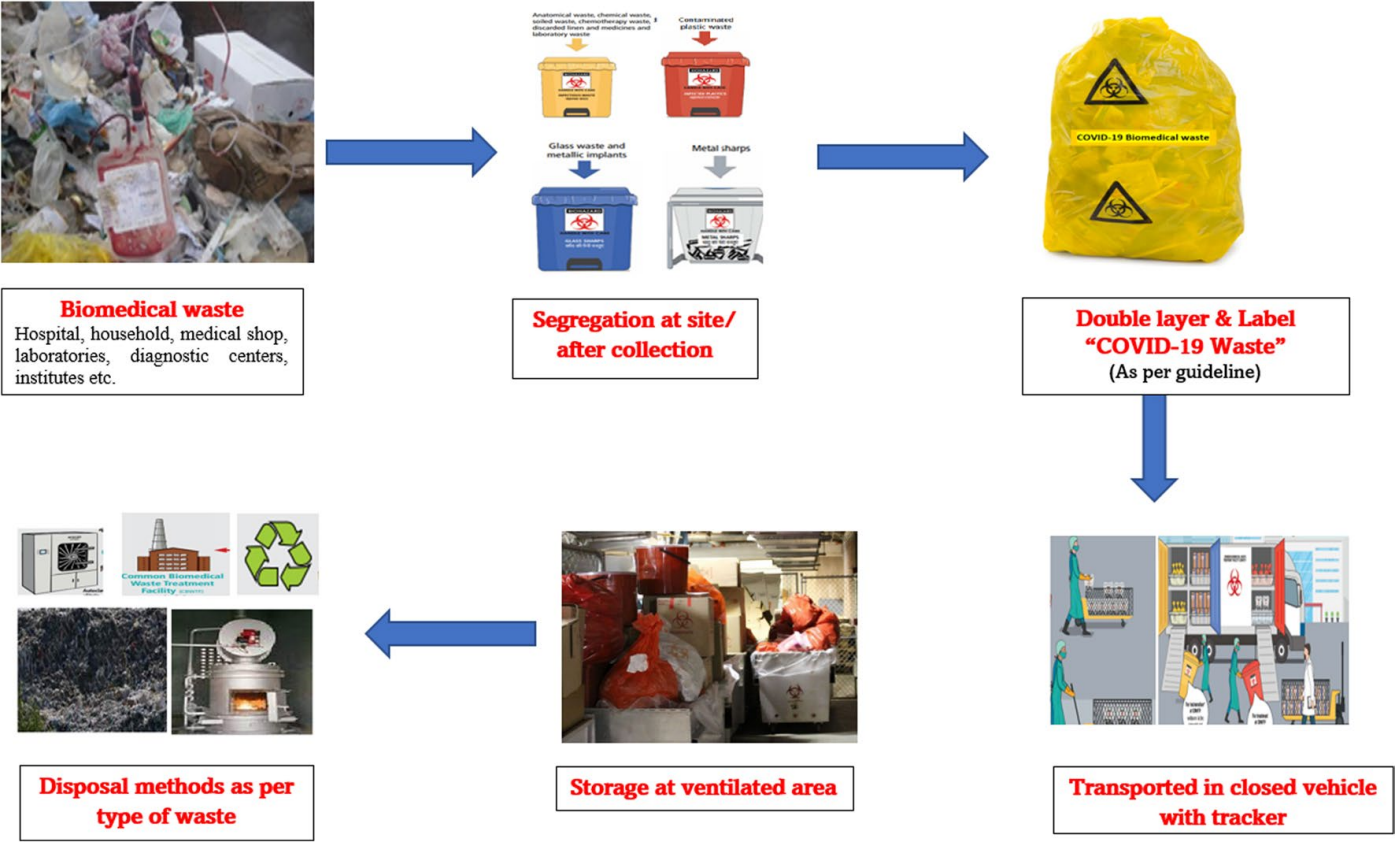
Guidelines by the CPCB for COVID-19 medical waste management

For pandemic COVID-19 condition, new rules and regulation are framed so that leakage should avoided by carrying the garbage in two safe containers along with handling the additional waste. The "COVID-19 Waste" label is affixed to the containers needed to handle and transport biomedical waste generated during the treatment of infectious diseases, in accordance with the guidelines of the Central Pollution Control Board (CPCB). However, the rules and regulations mentioned are mostly mandatory in hospitals, isolation wards, quarantine centers, and other medical centers. Experts are looking for standards and better waste management systems for disposing of COVID-19 medical waste at the household level. The figure 4 indicates the steps for COVID-19 medical waste management Central Pollution Control Board (2020a).

### Challenges in management of biomedical waste

- In all residential and public areas, bio-waste cannot be properly separated according to the color coding system in Appendix I (Schedule I) of the 1998 BMW Regulations or the 2016 Amended Regulations.
- Single-use surgical masks (along with N95 respirators) are frequently disposed of with general waste from homes and residential areas, which are not further packed in yellow color-coded BMW bins.
- There is no COVID-19 waste disposal standard system in existence for home quarantine centers.
- At quarantine centers, because of a lack of training (Basavaraj et al. 2021), awareness and manpower, people throw masks, PPE kits, gloves, and everything from food waste to disposable cutlery into yellow containers, which are then sent for incineration.
- The trolleys and containers which are used for transfer of biomedical waste are not used for any other function.
- Due to the short time span and lack of funds, the implementation of BMW rules 2016 is very difficult. Fund unavailability is the major lacuna in proper BMW management (Salman Zafar 2020).
- Segregation is manually carried out by waste handlers as well as they were not used complete protective equipment which can result in harm to health care workers.
- There are already 198 Common BMW treatment facilities (CBMWTF) in service in India, with another 28 under construction (Datta et al. 2018). India's requirement for a biomedical waste treatment and disposal facility includes an increase in number, modifications, and the development of novel disposable methods with high efficacy.
- Use of an outdated incinerator. It should be upgraded as per the ‘Central Pollution Control Board’ directions.
- The landfill method or deep burial of biomedical waste releases greenhouse gases causes’ groundwater contamination, the spread of infectious diseases, and the unavailability of land.
- Thermal heat can be generated from the process machinery like incinerators, boilers, etc. During incineration, thermal heat is generated, which is hazardous, and many times the material is not properly incinerated, making it much more toxic.

**Fig. 5:**
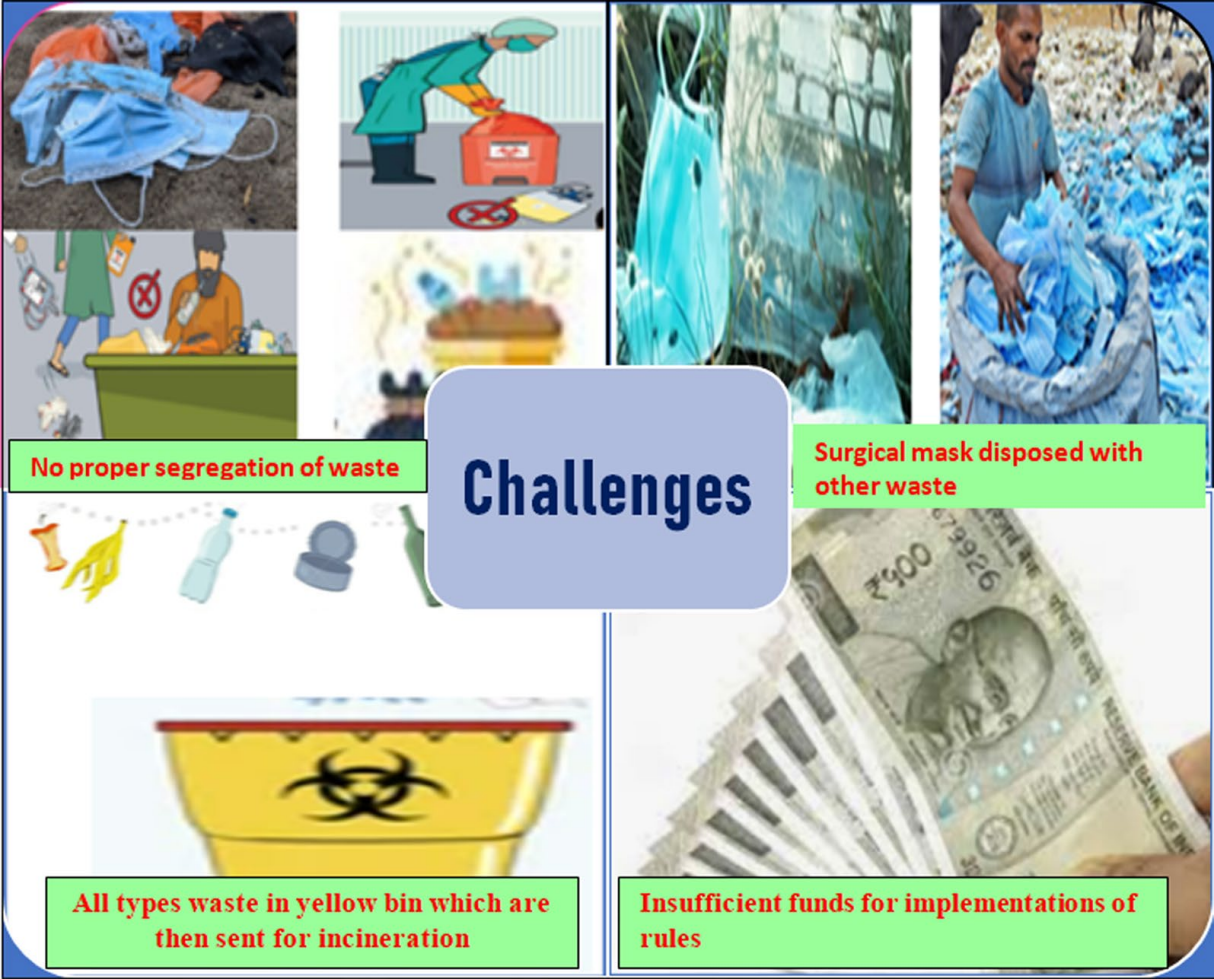
Challenges in management of COVID-19 biomedical waste

Figure 5 gives a short idea about challenges in COVID-19 biomedical waste management Central Pollution Control Board (2020d).

### Measures to handle the crisis of biomedical waste

- A potential short-term option to reduce waste generation is to reduce the use of PPE kits in non-COVID-19 locations and to use reusable masks and gloves.
- To reduce waste in quarantine camps and houses, use washable utensils.
- Waste separation at homes should be highly commended.
- Strengthening citizen participation and imposing separation at the source.
- Make sure all waste producers and disposers are registered with the COVID-19 app.
- Make sure that the vaccination camp outside the hospital is properly sorting waste.
- Proper training should be provided to BMW handlers for segregation, disposal, and self-safety precautions.
- As per Environment (Protection) Act, 1986 and other pollution control Acts strict action should be taken against the faulted hospitals/nursing homes.
- BMW should be collected from various centers only if they are properly segregated according to the color coded bag/container rule. It assists the waste collection vehicle.
- All BMW transport vehicles should be GPS equipped and tracked regularly.
- In favor of waste handlers, Safai Karamchari Ayog (non-statutory body that investigates the conditions of Safai Karamcharis (waste collectors) in India) demands a secure way to collect used masks and greater standard equipment for workers.
- Fill the deficiencies in the guidelines by updating them on a regular basis.
- CPCB should also consider remote area scenarios in terms of production and general practices for disposing of COVID-19 biomedical waste.
- The State Pollution Control Board (SPCB) needs to plan strategies that follow central agency guidelines to achieve integrity. Because they are responsible for pollution control and enforcement of control laws. Municipalities have introduced some waste management guidelines.
- According to the 2016 Biomedical Waste Disposal Regulations, bags used to collect biomedical waste must be bar-coded to track the source of waste that reaches the treatment facility. Kerala is one of the few states that have bar code garbage bags, which was an important part of the pandemic response.

### Consequences of negligence in biomedical waste management

BMW if not treated carefully, then it may be responsible for giving birth to large and various vectors that lead to the transmission of vector-borne diseases rapidly. It also causes pollution of land and water as well as through infected syringes and needles, the transmission of incurable diseases like AIDS and pandemic COVID-19.

BMW mixing with other garbage or poor BMW management causes air, water, and soil pollution. So, it gives birth to pathogenic diseases and poor health. Nowadays, per day generation of BMW and its treatment, it is inversely proportional. Such a situation indicates that soon our country will be drowning in its own garbage.

## Conclusions

Despite India's strict rules and regulations for the safe disposal of BMW, there is an instant need to take action to uplift the current system capacity, increase the funding and commitment toward the safe disposal of BMW. A sudden boost in BMW because of a pandemic condition should have optional measures for treatment of the generated biomedical waste. Innovative steps by the state pollution control board, along with initiation by the local municipal system, make it possible to get better control over BMW management. The Ministry of Environment, Forest, and Climate Change's Bio-Medical Waste Management Rules, 2016 and (Amendment) Rules, 2018 optimize separation, transit, and final treatment processes to decrease environmental contamination.

Finally, the talk is about the availability of funds. Adequate manpower and funds are required for the implementation of documented rules. Sanctioned funds pass through multiple government channels, and it takes a long time to reach the needy. So, by making the above-mentioned things easier India win the battle against BMW management and set an example for others.

## Data Availability

Literatures are collected from various resources such as Elsevier, Pubmed, Google scholar and various research journals.

## Abbreviations

BMW: Biomedical waste
CPCB: Central pollution control board
WHO: World health organisation
HCF: Health care facilities
TPD: Tones per day
COVIDBMW: COVID-19 biomedical waste
CSE: Centre for science and environment
PPE: Personal protective equipment
ULB: Urban local bodies
SPCB: State pollution control board
CBWTF: Common biomedical waste treatment facility
GPS: Global positioning system
AIDS: Acquired immune deficiency syndrome
APP: Application
